# Supra-Pubic Lithotomy, Etc.

**Published:** 1877-12

**Authors:** E. F. Starr

**Affiliations:** Nacoochee, Ga.


### SUPRA-PUBIC LITHOTOMY—A NEW OR UNUSUAL METHOD OF COMPLETING THE OPERATION, WITH A CASE.

By E. F. STARR, M.D., Nacoochee, Ga.

The history and literature of supra-pubic lithotomy indicate that comparatively little attention is bestowed upon this operation or method, while we know a vast amount of labor and inquiry have been given to other methods. To give relief from stone in the bladder has long been considered an important part of surgery, and since so much painstaking has been manifested to become possessed of the simplest, safest, best methods in most other surgical procedures, it seems a little singular that the high operation has not been more thoroughly investigated. But men are more or less like sheep—they follow their leader with a bound, whether wisely or unwisely, and many errors and misdirections have been perpetrated in this way. In the early days of lithotomy, operators failed to see and appreciate their opportunities for making this a safe, sightly and speedily successful operation. Some brought zeal and skill to bear on the lateral method, led their followers in that direction, ridiculed, misconstrued and misapplied the other, and so we may suppose the supra-pubic came to be regarded as a dernier resort, and not adapted to general use. It seems probable, too, that the disuse and neglect into which it fell, was, in a large measure, owing to the loose, open, unfinished and exposed manner in which the wound was left, thereby insuring a tedious recovery, if nothing worse.

"A large majority of the operations by this mode occurred at a period when surgery in all its branches was but imperfectly developed; when anæsthetics were unknown, and when there were few of the modern facilities for the cure of patients who have been subjected to so grave surgical injuries." (See article in American Journal of the Medical Sciences for July, 1875, by Dr. C. W. Dulles). And another remark by the same author serves further to illustrate how little thought has been given to it in the light of advanced medical knowledge. Dr. Dulles says, "The high operation is assigned a very low place in most works upon surgery, and is now so rarely practiced that there are comparatively few medical men who have ever seen it done; indeed, it has surprised me, in my investigations, to find how little is known of it by men of no inconsiderable eminence in the profession."

As Dr. Dulles has undertaken the history and defense of this operation, it is not my design to trespass upon his domain; but, while approving his labors, my object is to bring to notice a method of completing the operation by closing the wound, which, I think, will be found a great improvement upon the old way, and calculated to add much to the popularity of the operation.

In the American Journal of the Medical Sciences, for July, 1875, is an article by Dr. C. W. Dulles, of Philadelphia, entitled "Supra-pubic Lithotomy; an attempt to ascertain its merits and practicability as a general method, founded upon an analysis of 478 cases." Not long after the publication of this article, a case of stone in the bladder came into my hands, upon which I decided to practice the high operation. I did so for the following considerations: If results so favorable as this analysis shows, could follow such dernier resorts—that is, bad surgery and desperate cases—surely, quite satisfactory results might be expected from better surgery and more favorable cases; and that if the extraction of stone after the division of the important and complex tissues of the perineum, urethra, prostate gland and neck of the bladder may be done with reasonable success, a more complete success may be expected after the division of the simpler and less involved tissues of the walls of the lower abdomen, and of the front of the bladder, and that too, without the least possible risk (in the latter procedure) of producing impotency or incontinence of urine, which conditions may result from the lateral and bilateral operations.

Heretofore, it seems to have been the practice in supra-pubic lithotomy, to leave the wound in the abdomen, and sometimes even that in the bladder, *open*, to heal as best it might. And Dr. Dulles, in the article alluded to, recommends the bladder to be closed, but the outer wall to be left open yet stating that in the final process of healing, the bladder wall would become united to that of the abdomen. Now, if speedy and prompt healing and recovery is a desideratum after any wound, and if the bladder is to be united to the abdominal wall, then whatever course will most promote these ends is the best surgery.

With these views, I determined in the case in hand to close both wounds, and cause the adhesion of the two collectively by one process for all. By request, I reported this case to Dr. Dulles, and he published it, with remarks, in the July number of the *American Journal of the Medical Sciences* for 1877; but as there is an error in that publication in describing the sutures, which I wish to correct, I will reproduce the prominent parts of it, including the correction which I wish to make.

*Case.*—Mr. B. F. S., æt 35, a large, athletic man, had been an invalid for the last two or three years, but thought he had felt symptoms of stone for twelve years; for two or three years had been subject to occasional attacks of cystitis, setting in with shivering, and resulting in pyrexia and difficult micturition, which caused him a great deal of suffering; under treatment, he had for the last two months escaped these paroxysms and improved in general health, so that at the time of the operation he weighed two hundred pounds.

On the 6th of May, 1876, with the patient under chloroform, I proceeded to perform supra-pubic lithotomy. The patient was laid upon his back on a low couch, with his feet down and resting on the lower round of a chair, while I sat by his right side and made the first incision from above downward, and about four inches in length.

Some small arterial branches spouted a little blood, which was carefully sponged away, and before entering the pelvic cavity, further time was given for the general oozing to cease. After this, using the finger as a guide, the bladder wall was reached, the peritoneum being entirely avoided.

The feet were then elevated to the top of the couch, a sound introduced through the urethra, and the bladder raised up between the lips of the incision. The amount of fatty tissue enveloping the viscus made it somewhat difficult to denude, but a very clean dissection was not deemed indispensable, and when fairly cleared it was seized with a tenaculum, punctured just below with a sharp-pointed bistoury, and then incised to a sufficient extent with a Blizzard's knife upon a grooved director.

The bladder had been ordered to be emptied before the administration of the ænesthetic, but the kidneys being in a very active state from the previous free use of diuretic and demulcent drinks, and the time required to produce anæsthesia having been considerable, in addition to that consumed in making the incision, some urine had accumulated, and a part of it escaped through the external wounds, but as this was pretty well filled by the bladder as it was held up, the urine passed freely out.

The finger was then introduced, the stone found and brought out upon its palmar surface. It was smooth, of a flat, oval shape, and weighed one ounce and one drachm.

The thickness of the abdominal wall made the wound appear formidable, and its closure promise difficulty. This, however, was not so great as was anticipated.

At this point of the operation, I departed from the plan proposed in the *American Journal of Medical Science*, for July, 1875, and from former usage, and instead of closing the bladder alone by the lembert, or by the continued suture, I closed both the bladder and the parietes of the abdomen at once, by the interrupted suture, which was made to act somewhat as the lembert suture in relation to the bladder. That is to say, I passed a silver suture through the wall of the abdomen on one side, then made it to include a small portion of the coats of the bladder on the same side, then across the incision outside of the bladder, then into the coats of it again as before, on the opposite side, and thence through the abdominal wall, emerging at a point opposite to the point of entrance. When this suture was drawn, the wounds in both bladder and abdomen were closed, but the edges of the incised bladder were inclined inward or inverted, thus bringing the outer or serous surfaces in contact. In other words, the common interrupted suture was made to include all the tissues, but in relation to the bladder it was so inserted as to act as the lembert suture. The effects of this method of closure was to bring all the parts into close contact, prevent infiltration, promote speedy union, and secure quick recovery.

Drainage was instituted by keeping a small catheter in the urethra, and turning the outer end down into a cup between the legs, which was required to be removed and cleansed occasionally. At the expiration of ten days the cathetra was finally removed, and he got on his feet and passed his urine per urethram. At this time the remaining sutures were removed, except one, which was allowed to remain until the sixteenth day, (longer than necessary,) when it was taken away and the patient discharged, cured. During the whole time there was no inflammation nor swelling nor indication of burrowing or abscess about the wound, and it seems to me clear that this method of closing the wound is far preferable, both in point of time and safety, to that of leaving either one or both wounds open.

As to the merits of the high operation, as compared with other methods, it is my opinion that if ease, simplicity, safety and speedy recovery are to be regarded as substantial and available recommendations, then upon extended and impartial trial it must stand approved as equal, if not superior, to any or all other methods. But many are fond of old ways and old experiences, and are loth to quit the one or ignore the other; yet, if these influences and the love of show, parade and paraphernalia do not prevail to too great an extent, this operation is destined to emerge from its unmerited obscurity and take its position high among the nseful procedures of surgery.

